# Acute radiation skin injury in stage III-IV head and neck cancer: scale correlates and predictive model

**DOI:** 10.1186/s12957-024-03490-7

**Published:** 2024-07-25

**Authors:** Zihan Zhou, Junjian Lin, Ying Wang, Yunhao Chen, Yang Zhang, Xingchen Ding, Benhua Xu

**Affiliations:** 1 Department of Radiation Oncology, Fujian Medical University Union Hospital, Fuzhou, China; 2 Department of Radiation Oncology, Shandong Cancer Hospital and Institute, Shandong First Medical University and Shandong Academy of Medical Sciences, Jinan, Shandong China; 3 Department of Radiation Oncology, Jiangsu Cancer Hospital, Nanjing Medical University, Nanjing, Jiangsu China; 4 Department of Radiation Oncology, Shandong University Cancer Center, Jinan, Shandong China; 5 Fujian Key Laboratory of Intelligent Imaging and Precision Radiotherapy for Tumors (Fujian Medical University), Fuzhou, Fujian China; 6 Clinical Research Center for Radiology and Radiotherapy of Fujian Province (Digestive, Hematological and Breast Malignancies), Fuzhou, Fujian China

**Keywords:** Stage III-IV head and neck cancer, Acute radiation skin injury, Predictive factor

## Abstract

**Purpose:**

Active radiation skin injury (ARSI) has the highest incidence of acute adverse reactions caused by radiotherapy (RT) in patients with head and neck cancer (HNC). This study aimed to screen risk factors that can facilitate the identification of HNC patients at high risk of ARSI.

**Methods:**

Data from 255 stage III-IV HNC patients who underwent intensity-modulated radiation therapy (IMRT) were collected. The data from our medical records, including clinical characteristics and hematological indices before RT, were retrospectively collected and arranged. The Common Terminology Criteria for Adverse Events Criteria (CTCAE), Radiation Therapy Oncology Group Criteria (RTOG), World Health Organization Criteria (WHO), Oncology Nursing Society (ONS), Acute Radiation Dermatitis Graduation Scale, Douglas & Fowler and Radiation Dermatitis Severity Scale (RDSS) were used to assess ARSI. Of these, CTCAE was used for further analysis. Binary logistic regression analyses were used to identity risk factors. To establish the correction between each risk factor and the ARSI score, the odds ratio (OR) and 95% confidence interval (CI) were computed.

**Results:**

The assessment results of the CTCAE with RTOG, WHO, ONS, Graduation Scale, Douglas & Fowler and RDSS have good consistency. After radiotherapy, 18.4% of patients had at least 3 (3 +) grade ARSI. Multivariate logistic regression analysis revealed that the KPS score, blood glucose level, white blood cell count, and plasma free thyroxine (FT4) concentration were independent risk factors for 3 + grade ARSI. A nomogram was constructed on the basis of these risk factors, which demonstrated good predictive power according to the area under the ROC curve (AUC). The satisfactory consistency and clinical efficacy of the nomogram were confirmed by calibration curves and decision curve analysis (DCA).

**Conclusion:**

A low KPS score, high blood glucose level, high white blood cell count, and high thyroid hormone prior to radiotherapy for stage III-IV HNC are independent risk factors for grade 3 + RSI.

**Supplementary Information:**

The online version contains supplementary material available at 10.1186/s12957-024-03490-7.

## Introduction

The incidence of head and neck cancer (HNC), which include laryngeal cancers, oropharyngeal cancers, hypopharyngeal cancers and nasopharyngeal cancers et al., has increased to sixth among all tumors worldwide [1, 2]. Radiation therapy (RT) is a highly effective treatment strategy that substantially affects the management of HNC [3]. In addition, with the development of surgery, chemotherapy, targeted therapy and immunotherapy, the tumor control of patients with HNC has improved to a certain extent [4, 5]. More attention needs to be given to the adverse effects of radiotherapy.

Acute radiation skin injury (ARSI) is the most common complication associated with RT. Almost all HNC patients who received RT have severe damage to the skin. These injuries can result in significantly impaired quality of life, and severe toxicities may lead to treatment interruption, and even impair patient prognosis [6–8]. The TNM staging system is widely used in the prognostic assessment of cancer patients, but there is no universally accepted methodology for predicting ARSI. Consequently, developing clinically applicable tools for accurate prediction of the RSI in HNC patients before RT will aid in identifying groups at risk and delivering interventions in a timely manner.

Some previous studies revealed that clinical characteristics such as a lower Karnofsky Performance Scale (KPS) score, multicycle chemotherapy, and a skin dose volume > 50 Gy (V50) are associated with the RSI in HNC patients [9, 10]. The critical role of inflammation for normal tissue toxicity is indisputable [11]. Hence, the associated inflammatory indicators of blood tests are also necessary to analyze. A previous study revealed that ferritin, hs-CRP, and CD3 + T cells before RT are all independent risk factors for grade 4 + ONS ARSI in patients with breast cancer [12]. However, similar studies in HNC are still lacking. Furthermore, because different scales have been used in different studies to evaluate the severity of ARSI, the results of different studies are somewhat difficult to compare with each other.

In this study, we evaluated the consistency between the seven clinical scales and chose the CTCAE 5.0 scale, which is the most commonly used scale, to explore the predictors of ARSI. In addition, we developed an ARSI risk prediction nomogram for individualized risk assessment. With the use of a simplified visual model, a tight focus and interventions can be adopted in a timely manner for HNC patients receiving RT with high ARSI risk levels in the era of precision medicine.

## Methods

### Patients

**Fig. 1:**
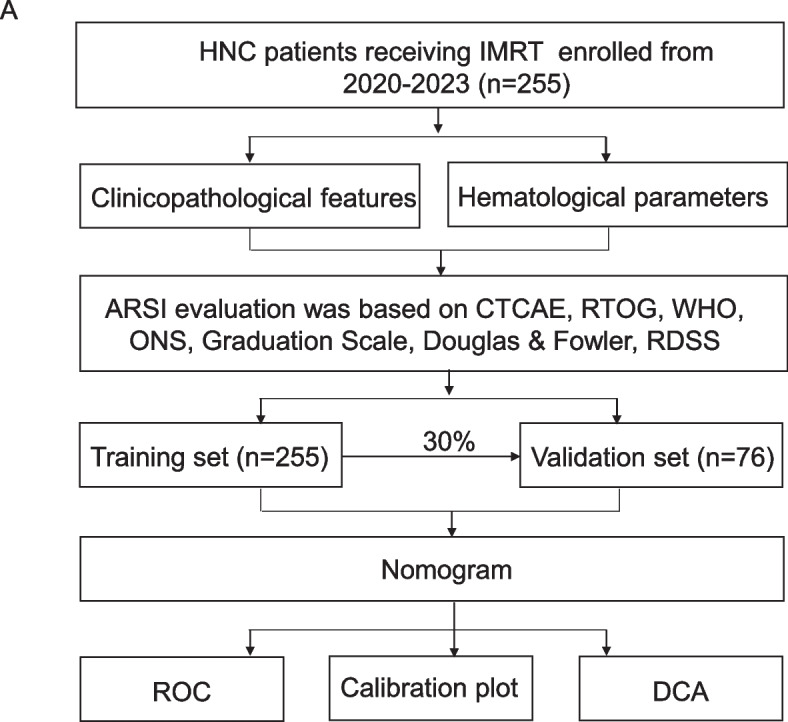
The study flowchart

During the period from May 24, 2020 to December 31, 2023, stage III-IV head and neck cancer patients receiving intensity-modulated radiation therapy (IMRT) were enrolled consecutively from Shandong Cancer Hospital. Informed consent was obtained from each patient to participate in the study. The inclusion criteria were as follows: (1) Age equal to or greater than 18 years, (2) Patients were newly diagnosed with stage III-IV HNC (nasopharynx, larynx, oropharynx, hypopharynx) according to eighth edition of the AJCC to identity the TNM staging system, (3) chemotherapy was performed concurrently with radiotherapy. The exclusion criteria were as follows: (1) double or multiple primary cancers; (2) previous irradiation, (3) skin disease or active knot-hoof tissue disease. The clinicopathological features and hematological parameters of patients before RT were collected from our institutional electronic medical record system. The study design is presented in Fig. 1.

### Treatment and RSI assessment

All patients received intensity-modulated radiotherapy (IMRT), and daily treatment with 2.0 Gy fractions. The total dose range was 60–70 Gy. The RT target area and organs at risk were delineated and defined according to the Radiation Therapy Oncology Group (RTOG) guidelines. RSI symptoms were recorded every 3 days during RT and one month after RT in the electronic medical records systems by the attending physician. The severity of the RSI was assessed on seven types of acute skin toxicity scales, including the Common Terminology Criteria for Adverse Events Criteria (CTCAE), Radiation Therapy Oncology Group Criteria (RTOG), World Health Organization Criteria (WHO), Oncology Nursing Society (ONS), Acute Radiation Dermatitis Graduation Scale, Douglas & Fowler and Radiation Dermatitis Severity Scale (RDSS) by the attending physician and the followers of this study. The final analysis was established by using the most severe dermal reaction during follow-up.

### Nomogram construction and validation

All 255 HNC patients formed the training cohort, and 76 (30%) patients were randomly selected as the validation cohort. The training cohort was used to construct the nomogram model via the following procedure. First, univariate logistic regression analysis was used to screen potential ARSI risk factors. Then variables with a P value less than 0.1 were incorporated into multivariate logistic regression to determine independent risk factors with P value less than 0.05 for ARSI. In this process, the odds ratio (OR) and 95% confidence interval (CI) were computed for each variable. A nomogram for predicting ARSI risk was constructed using all the independent risk factors identified via multivariate logistic regression. The validity of the nomogram model was examined in the following three ways. A nomogram for predicting ARSI risk was constructed using all the independent risk factors identified through multivariate logistic regression. The validity of the nomogram model was examined in the following three ways. The nomogram's ability to discriminate was estimated via the AUC, which represents the area under the ROC curve. A calibration plot and the Hosmer–Lemeshow (H–L) test were performed to assess the agreement between the estimated probability and actual probability. Decision curve analysis (DCA) diagrams measure the overall advantages of evaluating the practicality of the model.

### Statistical analysis

Correlations between different scales were analyzed via Sperman’s correlation. Both the chi-square test and Fisher's exact test were used for categorical variables. The optimal threshold for continuous values was identified by calculating the ROC curve and Youden index. All the data were computed via GraphPad Prism (version 10.1.2) and R software (version 4.2.3). A significance level of *P* < 0.05 for a two-tailed test was deemed statistically significant.

## Results

### Patient characteristics

Table 1 displays the clinical features of 255 individuals. There was no notable distinction observed between the training and validation groups (all P-values were greater than 0.05). In the training and validation cohorts, the percentages of patients rated as grade 3 + (CTCAE 5.0) were 18.43% and 19.74%, respectively, with no significant difference (*P* = 0.798). The time from the beginning of RT to the occurrence of skin injury was 20.256 ± 8.329 days (range, 5–65 days).

**Table 1:** Patient characteristics

Variable n (%)	Training cohort (*n* = 255)	Validation cohort (*n* = 76)	P
Gender			0.595
Male	226 (88.63)	69 (90.79)	
Female	29 (11.37)	7 (9.21)	
Age			0.590
< 60	163 (63.92)	46 (60.53)	
≥ 60	92 (36.08)	30 (39.47)	
KPS			0.794
< 90	71 (27.84)	20 (26.32)	
≥ 90	184 (72.16)	56 (73.68)	
Smoking			0.551
Yes	131 (51.37)	42 (55.26)	
No	124 (48.63)	34 (44.74)	
Alcohol			0.301
Yes	117 (45.88)	40 (52.63)	
No	138 (54.12)	36 (47.37)	
BMI			0.940
< 25	209 (81.96)	62 (81.58)	
≥ 25	46 (18.04)	14 (18.42)	
Coronary Artery Disease			0.916
Yes	13 (5.10)	3 (3.95)	
No	242 (94.90)	73 (96.05)	
Hypertension			0.866
Yes	56 (21.96)	16 (21.05)	
No	199 (78.04)	60 (78.95)	
Diabetes			0.811
Yes	14 (5.49)	3 (3.95)	
No	241 (94.51)	73 (96.05)	
T			0.505
1–2	95 (37.85)	32 (42.11)	
3–4	156 (62.15)	44 (57.89)	
N			0.884
0–1	62 (24.51)	18 (23.68)	
2–3	191 (75.49)	58 (76.32)	
TNM stage			0.583
III	82 (32.2)	27 (35.5)	
IV	173(67.8)	49 (64.5)	
Targeted drug			0.960
Yes	16 (6.27)	4 (5.26)	
No	239 (93.73)	72 (94.74)	
WBC			0.709
Low	127 (49.80)	36 (47.37)	
High	128 (50.20)	40 (52.63)	
Neu			0.820
Low	117 (45.88)	36 (47.37)	
High	138 (54.12)	40 (52.63)	
RBC			0.983
Low	215 (84.31)	64 (84.21)	
High	40 (15.69)	12 (15.79)	
HGB			0.752
Low	43 (16.86)	14 (18.42)	
High	212 (83.14)	62 (81.58)	
Lym			0.137
Low	152 (59.61)	38 (50.00)	
High	103 (40.39)	38 (50.00)	
PLT			0.316
Low	107 (41.96)	27 (35.53)	
High	148 (58.04)	49 (64.47)	
ALB			0.628
Low	239 (93.73)	73 (96.05)	
High	16 (6.27)	3 (3.95)	
TG			0.877
Low	164 (64.82)	50 (65.79)	
High	89 (35.18)	26 (34.21)	
BG			0.484
Low	185 (72.55)	52 (68.42)	
High	70 (27.45)	24 (31.58)	
FT4			0.467
Low	20 (7.87)	8 (10.53)	
High	234 (92.13)	68 (89.47)	
CTCAE			0.798
0–2	208 (81.57)	61 (80.26)	
3–4	47 (18.43)	15 (19.74)	

### Correlation analysis and comparative analysis of different scales

**Fig. 2:**
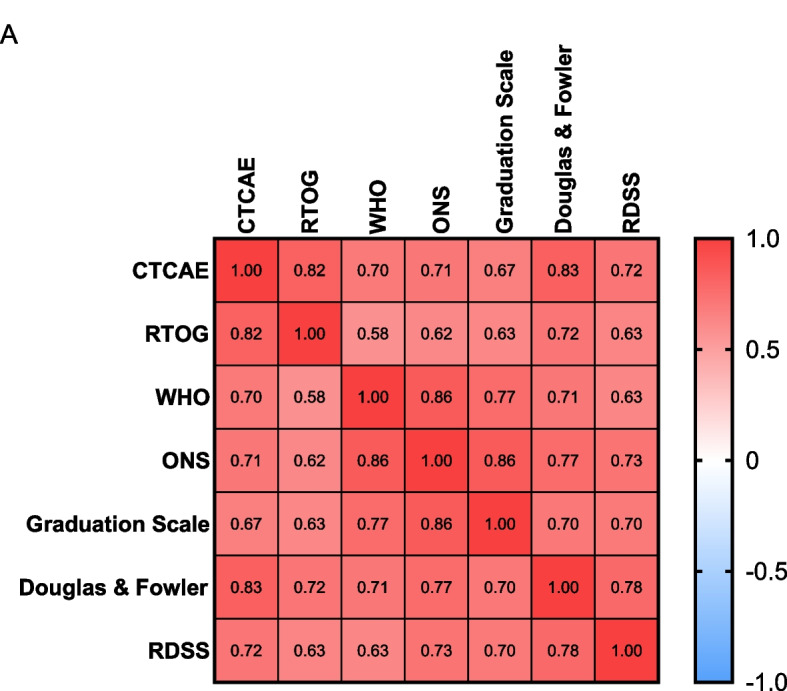
Correlations between different scales

Spearman correlation analysis of the CTCAE with the RTOG, WHO, ONS, Graduation Scale, Douglas & Fowler and RDSS scores revealed highly positive correlations, and the correlation coefficient was between 0.67 and 0.86, indicating the consistency of the CTCAE with the RTOG, WHO, ONS, Graduation Scale, Douglas & Fowler and RDSS assessment results (Fig. 2A).

### Univariate and multivariate analyses

To achieve more parsimonious models to predict the risk of Grade 3 + ARSI, an ROC curve was used to determine the best cutoff point for the WBC count, white blood cells (5.875 10^9^/L); Neu, neutrophils (3.48 10^9^/L); RBC, red blood cells (4.85 10^12^/L); HGB, hemoglobin (107.5 g/L); Lym, lymphocytes (1.585 10^9^/L); PLT, platelets (220 10^9^/L); FT4, free tetraiodothyroxine (17 pmol/L); ALB, albumin (49.25 g/L); TG, triglycerides (1.4 mmol/L); and BG, blood glucose (5.55 mmol/L). Patients were divided into lower-level and higher-level groups. A univariate analysis was conducted to screen risk factors for grade 3 + (CTCAE 5.0) ARSI. Table 2 indicates that a lower KPS, the use of targeted drugs, and higher WBC, Neu, Lym, TG, BG and FT4 values were statistically associated with a greater risk of a 3 + RSI.

**Table 2:** Univariate and multivariate analysis in predicting grade 3 + ARSI

Variable	Univariate analysis			Multivariate analysis		
	OR	95%CI	P	OR	95%CI	P
Gender (Male vs. Female)	1.574	0.628–3.947	0.333			
Age (< 60 vs. ≥ 60)	1.093	0.562–2.126	0.794			
KPS (< 90 vs. ≥ 90)	0.392	0.203–0.756	**0.005**	0.223	0.069–0.723	**0.012**
Smoking (No vs. Yes)	0.985	0.523–1.855	0.963			
Alcohol (No vs. Yes)	1.290	0.685–2.432	0.431			
BMI (< 25 vs. ≥ 25)	1.291	0.589–2.832	0.524			
Coronary Artery Disease (Yes vs. No)	0.741	0.196–2.803	0.659			
Hypertension (Yes vs. No)	0.682	0.331–1.403	0.298			
Diabetes (Yes vs. No)	1.378	0.298–6.372	0.682			
T (1–2 vs. 3–4)	1.092	0.565–2.111	0.792			
N (0–1 vs. 2–3)	1.463	0.663–3.226	0.346			
TNM stage (III vs. IV)	0.901	0.461–1.760	0.759			
Target drug (Yes vs. No)	0.195	0.069–0.551	**0.002**			
WBC (Low vs. High)	1.981	1.030–3.811	**0.041**	4.083	1.219–13.680	**0.023**
Neu (Low vs. High)	2.312	1.169–4.572	**0.016**			
RBC (Low vs. High)	0.444	0.150–1.316	0.143			
HGB (Low vs. High)	1.196	0.496–2.883	0.690			
Lym (Low vs. High)	2.106	1.110–3.996	**0.023**			
PLT (Low vs. High)	1.507	0.777–2.923	0.225			
ALB (Low vs. High)	1.610	0.494–5.241	0.429			
TG (Low vs. High)	2.264	1.190–4.306	**0.013**			
BG (Low vs. High)	2.341	1.210–4.528	**0.012**	3.950	1.243–12.548	**0.020**
FT4 (Low vs. High)	4.062	1.406–11.740	**0.010**	3.898	1.195–12.714	**0.024**

A multivariate logistic regression analysis was performed, including KPS, the use of targeted drugs, WBC, Neu, Lym, TG, BG and FT4. In the multivariate logistic regression (Table 2), a lower KPS (OR: 0.223, 95% CI: 0.069–0.723, *P* = 0.012), and higher WBC (OR: 4.083, 95% CI: 1.219–13.680, *P* = 0.023), BG (OR: 3.950, 95% CI: 1.243–12.548, *P* = 0.020) and FT4 (OR: 3.898, 95% CI: 1.195–12.714, *P* = 0.024) were independent prognosticators of the 3 + grade RSI.

### Development and validation of the nomogram

**Fig. 3:**
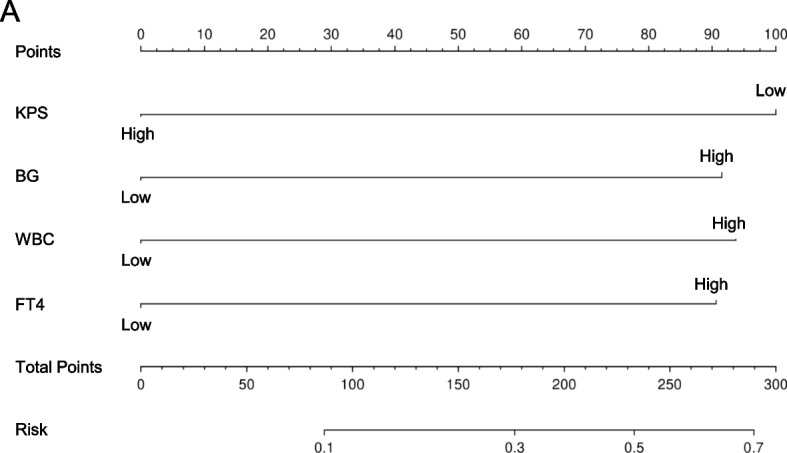
Nomogram predicting the risk of grade 3 + RASI

**Fig. 4:**
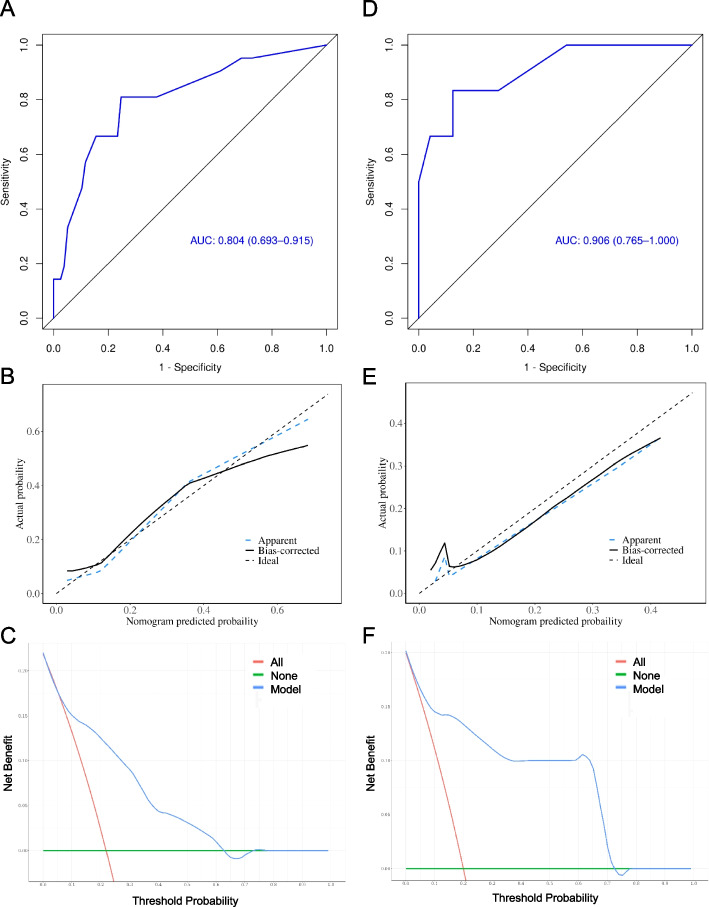
ROC curves, calibration curves and DCA of the nomogram predicting the risk of grade 3 + ARSI in training cohort (**A**-**C**) and validation cohort (**D**-**F**), respectively

A grade 3 + nomogram model based on KPS, WBC, BG and FT4, was constructed via multivariate logistic analysis (Fig. 3A). Compared with KPS (0.603; 95% CI, 0.526–0.681), WBC (0.584; 95%CI, 0.506–0.661), BG (0.598; 95% CI, 0.519–0.676) and FT4 (0.636; 95% CI, 0.521–0.752) alone, the AUC of the nomogram has exceptional performance (0.804; 95% CI, 0.693–0.915) in identifying grade 3 + RSI (Fig. 4A). The calibration curves revealed satisfactory consistency between the prediction of grade 3 + ARSI and the actual observation, and the H–L test for the nomogram indicated an adequate fit (*P* value greater than 0.05). (Fig. 4B). DCA revealed good positive net benefits at the threshold probabilities (Fig. 4C). Furthermore, the 30% training cohort underwent internal validation. The AUC of the nomogram (0.906; 95% CI, 0.765–1.000) remained superior to that of any other single predictor (KPS: 0.627, 95% CI, 0.486–0.767; WBC: 0.587, 95% CI, 0.449–0.726; BG: 0.619, 95% CI, 0.476–0.762; FT4: 0.604, 95% CI, 0.387–0.822), demonstrating its stronger ability to differentiate 3 + grade ARSI (Fig. 4D). Calibration curves and DCA further confirmed the accuracy and clinical feasibility of the nomogram (Fig. 4E-F).

## Discussion

In this study, the KPS, the use of targeted drugs, WBC count, Neu, Lym, TG, BG and FT4 before RT were found to be predictors of grade 3 + ARSI in stage III-IV HNC patients undergoing RT.

The KPS score reflects the health status and ability of patients to receive treatment [13]. Patients with al lower KPS often experience worse toxicity control [14]. Zexin Yao et al. reported that the KPS score is a significant patient-specific risk factor for radiation-induced skin lesions in patients with nasopharyngeal carcinoma [9]. This finding is consistent with our results.

This study revealed that the use of targeted agents can aggravate skin injury. The targeted agents include Cetuximab used in HNSCC and Nimotuzumab used in NPC, which all belong to Anti-EGFR target therapies. To date, whether anti-EGFR targeted therapy can aggravate the RSI remains controversial. Some studies show that the Anti-EGFR target therapies does not significantly increase the RT-related adverse reactions [15, 16]. Some reported greater significantly higher frequency of radiation skin injury [17–20]. In terms of mechanism, epithelial growth factor receptor (EGFR) plays important roles in both epidermal development and maintenance and in inflammatory and immune responses. Anti-EGFR targeted therapies can inhibit cell proliferation and migration, and improve sensitivity to RT [21, 22]. These effects synergize and generate greater intensity, frequency reaction in shorter, which can finally result in skin necrosis. Simultaneously, they produce more inflammatory exudate, aggravate inflammation, and increase the risk of infection [23].

Inflammation may be one of the key factors in skin injury. On the one hand, RT can recruit inflammatory cells to injured skin tissue directly. On the other hand, proinflammatory cytokines and growth factors are activated, which promotes inflammation and cytokine overproduction [11]. WBCs are the hallmark cells of the body’s inflammatory response and are classified into neutrophils, lymphocytes, basophils, eosinophils, and monocytes [24]. Among these types, neutrophils and lymphocytes are the most abundant types [25]. Neutrophil migration is a hallmark of inflammation, and the inflammatory infiltrate is predominantly lymphocytic [26, 27]. Our study revealed that higher WBC, Neu and Lym values were statistically associated with a greater risk of 3 + ARSI, which may reflect the inflammatory stress state in patients before RT.

In this study, we found that high TG and BG levels were associated with severe skin injury. We did not find direct evidence of how the TG and BG regulate ARSI. However, one previous study reported that statins play important roles in accelerating DNA repair in vascular smooth muscle cells [28]. In addition, another study revealed that statins (antilipidemic drugs) reduce the mRNA expression of RT-induced proinflammatory and profibrotic cytokines in vitro and alleviate inflammation and fibrosis stimulated by RT in vivo [29]. Maysa Pouri et al. reported that gliclazide (antidiabetic drug) is a powerful radioprotective agent that can protect healthy cells from RT-induced chromosome damage via its antioxidant activity [30]. Mohsen Cheki et al. demonstrated that metformin, an effective radioprotector, can reduce RT-induced DNA damage and apoptosis in human lymphocytes [31]. The results of these studies suggest that reducing blood sugar and blood lipids may enhance the protective effect of RT. Conversely, high TG and BG levels may exacerbate skin injury.

The activation of the inflammatory response is closely linked with the activation of innate immunity [32]. Thyroid hormones are involved in the regulation of innate immune responses, which may have the following effects: (1) T4 induced respiratory-burst activity and stimulated MPO activity in human polymorphonuclear leukocytes (PMNLs) [33]. (2) T4 supplementation increased the expression of costimulatory molecules in DCs [34, 35]. (3) T4 boosted IFNγ and IL-2 response in NK cells [36–38]. (4) A stimulatory effect of T4 on the phagocytosis process of cultured peritoneal mouse macrophages has been reported [39, 40].

In this study, the methodological approach used to predict the ARSI is simple. We developed a nomogram developed on the basis of clinical characteristics and blood test indicators to predict the risk of severe ARSI in individual patients, allowing for individualized risk prediction. Several limitations of this study should be discussed. First, as this was a small sample size and single-center study, selection bias might be present. Second, we explored only the clinical characteristics and hematological markers commonly measured in the clinic for the prediction of ARSI. However, new technologies such as dermoscopic and ultrasonographic examinations have become increasingly common. Therefore, integrating much more information from multimodal medical imaging might further optimize the performance of the prediction model.

### Supplementary Information

Supplementary Material 1.

## Data Availability

No datasets were generated or analysed during the current study.
