# Supplementary material for: Acute radiation skin injury in stage III-IV head and neck cancer: scale correlates and predictive model
Source: World J Surg Oncol. 2024 Jul 25;22:195. doi: 10.1186/s12957-024-03490-7 (PMC11271214; doi:10.1186/s12957-024-03490-7)
Supplement: Supplementary file 1 — Supplementary Material 1. [file 12957_2024_3490_MOESM1_ESM.docx]

**Common terminology criteria for adverse events (****CTCAE)**

| **Grade 1** | **Grade 2** | **Grade 3** | **Grade 4** | **Grade 5** |
| --- | --- | --- | --- | --- |
| faint erythema or dry desquamation | moderate to brisk erythema, patchy moist desquamation, mostly confined to skin folds and creases, moderate edema | moist desquamation in areas other than skin folds and  creases, bleeding induced by minor trauma or abrasion | life-threatening consequences, skin necrosis or ulceration of full thickness dermis, spontaneous bleeding from involved site, skin graft indicated. | death |

**Radiation Therapy Oncology Group Criteria (RTOG)**

**Acute** **Radiation Dermatitis (original version)**

| **Grade 0** | **Grade 1** | **Grade 2** | **Grade 3** | **Grade 4** |
| --- | --- | --- | --- | --- |
| No change over baseline | Follicular, faint or dull erythema/epilation/dry desquamation/decreased sweating | Tender or bright erythema, patchy moist desquamation/moderate edema | Confluent, moist desquamation other than skin folds, pitting edema | Ulceration, hemorrhage, necrosis |

**World Health Organization Criteria (WHO)**

| **Grade 0** | **Grade 1** | **Grade 2** | **Grade 3** | **Grade 4** |
| --- | --- | --- | --- | --- |
| None | Erythema | Dry desquamation, vesiculation, pruritus | Moist desquamation, ulceration | Exfoliative dermatitis, necrosis requiring surgical intervention |

**Oncology Nursing Society (ONS)**

| **Score** | **Observation** |
| --- | --- |
| 0 | No change |
| 1.0 | Faint or dull erythema |
| 1.5 | Bright erythema |
| 2.0 | Dry desquamation with or without erythema |
| 2.5 | Small to moderate amount of moist desquamation |
| 3.0 | Confluent moist desquamation |
| 3.5 | Ulceration, hemorrhage, or necrosis |

**Acute Radiation Dermatitis Graduation Scale**

| Grade | Description |
| --- | --- |
| 0 | no change |
| 1 | hyperpigmentation |
| 2 | erythema |
| 3 | skin dryness |
| 4 | dry desquamation located in one or more separate points |
| 5 | dry desquamation disseminated in one or more adjacent points |
| 6 | local moist desquamation and/or in folds |
| 7 | disseminated moist desquamation |
| 8 | Bleeding and/or ulceration |
| 9 | Necrosis |

**Douglas & Fowler**

| **Score** | **Observation** |
| --- | --- |
| 0 | Normal |
| 0.25 | 50/50, Doubtful if any difference from normal |
| 0.5 | Very slight reddening |
| 0.75 | Definite but slight reddening |
| 1 | Severe reddening |
| 1.25 | Severe reddening with white scale; ‘‘papery’’ appearance of skin |
| 1.5 | Moist breakdown in one very small area with scaly or crusty appearance |
| 1.75 | Moist desquamation in more than one small area |
| 2 | Moist desquamation in 25% of irradiated area |
| 2.25 | Moist desquamtion in 33% of irradiated area |
| 2.5 | Moist desquamation in 50% of irradiated area |
| 2.75 | Moist desquamation in 66% of irradiated area |
| 3 | Moist desquamation in most of irradiated area |
| 3.25 | Moist desquamation in most of irradiated area with slight moist exudate |
| 3.5 | Moist desquamation in most of irradiated area with moist exudates; necrosis |

**Radiation dermatitis severity scale**

| **Score** | **Observation** |
| --- | --- |
| 0.0 | Normal or none |
| 0.5 | Patchy faint/slight follicular eyrthema; faint hyperpigmentation |
| 1.0 | Faint and diffuse erythema; diffuse hyperpigmentation; mild epilation |
| 1.5 | Definite erythema; extreme darkening/hyperpigmentation |
| 2.0 | Definite erythema/hyperpigmentation with fine dry desquamation; mild edema |
| 2.5 | Definite erythema/hyperpigmentation with branny/scaly desquamation |
| 3.0 | Deep red erythema with diffuse dry desquamation; peeling in sheets |
| 3.5 | Violaceous erythema with early moist desquamation; peeling in sheets; patchy crusting |
| 4.0 | Violaceous erythema with diffuse moist desquamation; patchy crusting; ulceration; necrosis |
